# Non-Antibiotics Strategies to Control *Salmonella* Infection in Poultry

**DOI:** 10.3390/ani12010102

**Published:** 2022-01-01

**Authors:** José Martín Ruvalcaba-Gómez, Zuamí Villagrán, Juan José Valdez-Alarcón, Marcelino Martínez-Núñez, Lorena Jacqueline Gomez-Godínez, Edmundo Ruesga-Gutiérrez, Luis Miguel Anaya-Esparza, Ramón Ignacio Arteaga-Garibay, Angélica Villarruel-López

**Affiliations:** 1 National Center for Genetic Resources, National Institute of Forestry, Agriculture and Livestock Research, Boulevard de la Biodiversidad 400, Jalisco 47600, Mexico; ruvalcaba.josemartin@inifap.gob.mx (J.M.R.-G.); gomez.lorena@inifap.gob.mx (L.J.G.-G.); 2 Los Altos University Center, University of Guadalajara, Av. Rafael Casillas Aceves 1200, Jalisco 47600, Mexico; blanca.villagran@academicos.udg.mx (Z.V.); edmruesga@gmail.com (E.R.-G.); luis.aesparza@academicos.udg.mx (L.M.A.-E.); 3 Multidisciplinary Center for Biotechnology Studies, Centenary and Meritorious University of Michoacán of San Nicolás de Hidalgo, Michoacán 58893, Mexico; jose.alarcon@umich.mx; 4 College of Postgraduates, Montecillo Campus, Mexico City 56230, Mexico; marcelino_ipn@hotmail.com; 5 University Center for Exact and Engineering Sciences, University of Guadalajara, Blvd. Marcelino García Barragán 1421, Jalisco 44430, Mexico

**Keywords:** antibiotics, *Salmonella*, probiotics, prebiotics, poultry, vaccines

## Abstract

**Simple Summary:**

This review is focused on describing the main available antibiotic-free strategies that may be implemented to control or reduce the impact associated with *Salmonella* infection in poultry. These alternatives have been cataloged in two groups: feeding-based (prebiotics, probiotics, synbiotics, postbiotics, and phytobiotics) and non-feeding-based strategies (bacteriophages, in ovo applications, and vaccines). Moreover, we highlighted the relevance of the omics as a tool to design and validate the effects and efficacy of these kinds of treatments when *Salmonella* control is pursued.

**Abstract:**

*Salmonella* spp. is a facultative intracellular pathogen causing localized or systemic infections, involving economic and public health significance, and remains the leading pathogen of food safety concern worldwide, with poultry being the primary transmission vector. Antibiotics have been the main strategy for *Salmonella* control for many years, which has allowed producers to improve the growth and health of food-producing animals. However, the utilization of antibiotics has been reconsidered since bacterial pathogens have established and shared a variety of antibiotic resistance mechanisms that can quickly increase within microbial communities. The use of alternatives to antibiotics has been recommended and successfully applied in many countries, leading to the core aim of this review, focused on (1) describing the importance of *Salmonella* infection in poultry and the effects associated with the use of antibiotics for disease control; (2) discussing the use of feeding-based (prebiotics, probiotics, bacterial subproducts, phytobiotics) and non-feeding-based (bacteriophages, in ovo injection, vaccines) strategies in poultry production for *Salmonella* control; and (3) exploring the use of complementary strategies, highlighting those based on -omics tools, to assess the effects of using the available antibiotic-free alternatives and their role in lowering dependency on the existing antimicrobial substances to manage bacterial infections in poultry effectively.

## 1. Introduction

*Salmonella* infections remain one of the most critical public health problems worldwide. According to the Center for Disease Control and Preventions (CDC), only in the United States of America, *Salmonella* causes 1.35 million infections per year, with diarrhea, fever, and abdominal pain as the main symptoms [1]. The presence of 7–8 log_10_ of *Salmonella* is required for the disease to develop, which generally consists of gastroenteritis that is usually self-limiting [2]. However, it can also cause extraintestinal infections, particularly in immunocompromised people [3]. For humans, the primary source of infection is poultry products (meat and eggs), often from healthy animals [4,5]. *Salmonella* transmission occurs horizontally and vertically in birds, causing a subclinical disease or not causing any alteration, which increases the possibility of zoonotic transmission to humans through the food chain [6,7]. Although it is unknown with certainty how *Salmonella* remains and spreads on farms, biofilm formation is one of the proposed strategies [8]. These biofilms can involve multiresistant strains to antibiotics and other factors that favor their permanence in the environment [9,10]. Therefore, in broilers, the reduction of *Salmonella* from the farm is essential to contribute to food safety. Due to this, the poultry industry has sought new strategies to control the presence of *Salmonella* in the poultry production chain, which could be classified as feeding- and non-feeding-based strategies. Among these strategies is the addition of probiotics [11], prebiotics [12], postbiotics, such as some bacteriocins [13], and other compounds such as phytobiotics [14] throughout the diet, which also promote food efficiency, acting as growth promoters. On the other hand, bacteriophages [15,16], in ovo applications [17,18], and vaccines [19,20,21] are viable and technological non-feeding-based strategies extensively proved and implemented to reduce or control *Salmonella* infection in poultry. Currently, -omic technologies can be used as complementary tools in poultry to obtain information that can result in the formulation of therapeutic strategies and for detecting patterns of resistance to antibiotics, reducing the presence of *Salmonella* and production costs [22].

This review summarizes and discusses the main available antibiotic-free strategies for *Salmonella* control in poultry and their efficiency in preventing *Salmonella* infection and reducing its adverse effects, besides exploring complementary approaches based on the *-omics* as a tool to their assessment.

## 2. Materials and Methods

### Sources of the Data and Search Strategy

This study aimed to review the available reports on the use of antibiotic-free strategies for *Salmonella* control in poultry, focusing on the feeding- and non-feed-based strategies. For this, a comprehensive search was performed online through Web of Science, PubMed, and SCOPUS databases. The inclusion criteria were articles where the authors applied antibiotic-free strategies (use of prebiotics, probiotics, synbiotics, postbiotics, phytobiotics, bacteriophages, in ovo applications, and vaccines) to control *Salmonella* infections in challenged laying hens, broilers, turkeys, and quails. The period of publication was from 2015 to 2021; however, publications in scoping (<2015) were considered for the review. In the present narrative review, all retrieved publications that met the inclusion criteria were considered (original, narrative review, bibliometric, systematic, meta-analysis, and editor letters).

## 3. The Genus *Salmonella* and Its Relevance in Poultry

The genus *Salmonella* corresponds to an enteric Gram-negative, facultative anaerobe and non-spore-forming bacillus with cell diameters ranging from 0.7 to 1.5 µm and lengths from 2 to 5 µm, that belongs to the *Enterobacteriaceae* family. They are chemotrophs and frequently have peritrichous flagella, except for *S.* Gallinarum and *S.* Pullorum, which are non-motile and severely pathogenic to poultry [23]. *Salmonella* is able to colonize and multiply under several environmental conditions outside of a living host cell and is considered a non-fastidious microorganism. Members of the *Salmonella* genus grow under temperatures from 7 to 48 °C, tolerating growing at water activity levels up to 0.995 and pH values between 6.5 to 7.5 [24].

The genus *Salmonella* is comprised of two species (based on the sequence analysis differences): *Salmonella enterica* and *Salmonella bongori*. The latter group is divided into six subspecies; meanwhile, *Salmonella enterica* comprises more than 2500 serovars, and about 80 of them have been commonly associated with salmonellosis in both animals and humans. On the other hand, *Salmonella bongori* comprises at least 20 serotypes and is commonly associated with cold-blooded animals, but it can also infect humans [25].

*Salmonella* infection in poultry has long been categorized as a zoonotic disease of economic importance in public health worldwide [7,26,27,28], for which poultry and poultry products have been considered as the major reservoir of *Salmonella,* with approximately 200 serovars isolated from them, being *Salmonella* Enteritidis and *Salmonella* Typhimurium the most identified serovars related to poultry outbreaks [29,30,31,32,33].

## 4. Feeding-Based Strategies to Control *Salmonella* Infection in Poultry

Over the last years, non-antibiotic alternatives to reduce or control *Salmonella* infections in poultry have been investigated, which are focused on the use of feeding-based strategies, including prebiotics, probiotics, synbiotics, postbiotics, and phytobiotics.

### 4.1. Prebiotics

The International Scientific Association for Probiotics and Prebiotics (ISAPP) defined *prebiotics* as “a substrate that is selectively utilized by host microorganisms conferring a health benefit” [34]. The term prebiotics includes some carbohydrates and related compounds, such as galactooligosaccharides (GOS), mannan-oligosaccharides (MOS), and fructooligosaccharides (FOS), which, after ingestion, are digested by the host or by gut-related microbiota (mostly lactic acid bacteria and bifidobacteria). Therefore, prebiotics are usually administered to induce a modulatory effect on the intestinal microbiota by enhancing the growth of resident beneficial bacteria [35,36,37]. Table 1 summarizes the dietary supplementation of prebiotics in poultry to prevent or control *Salmonella* infections.

Several authors have demonstrated the potential of prebiotics to reduce the incidence of *Salmonella* and reduce its adverse effects on the gastrointestinal tract of poultry. The supplementation of prebiotics such as FOS, *Aspergillus* meal, or trehalose significantly reduces cecal *Salmonella* and its horizontal transmission. This beneficial effect is attributable to the ability of prebiotics to modulate the gut microbiota [40], promoting the expression of molecules such as the toll-like receptor (TLR-4), associated with resistance to *Salmonella* infection. Furthermore, the administration of prebiotics increases the accumulation of IgA+ cells on the intestinal mucosa, which prevents *Salmonella* colonization [38,39]. Remarkably, *Aspergillus* meal reduces *Salmonella* colonization due to the synergistic effect with beta-glucan, MOS, chitosan, and FOS present in the mycelium of fungi [33]. Thus, the administration of prebiotics to poultry promotes the modulation of the gastrointestinal microbiota and subsequently triggers the needed mechanisms to inhibit the infection and horizontal transmission of *Salmonella*.

### 4.2. Probiotics

Probiotics are defined as “living microorganisms that, when administered in sufficient amounts, confer a health benefit to the host” [43]. Their inclusion as dietary supplements in poultry offers beneficial effects associated with their ability to inhibit the growth of pathogenic bacteria [44]. In this context, some probiotic bacteria (alone or combined) have been used to prevent or control *Salmonella* infections during poultry production. Scientific reports have demonstrated that dietary supplementation based on probiotics can improve productive performance [45], as well as prevent *Salmonella* infections and reduce their related negative effects [46]. Most of the bacteria used as probiotics for poultry supplementation include several species of *Bifidobacterium*, *Lactobacillus*, and *Bacillus.* Moreover, other genera, such as *Enterococcus* and *Pediococcus*, have been included. The main demonstrated effects of probiotics supplementation in poultry are related to the ability to restore the gut microbiota, especially in *Salmonella*-challenged laying hens, as well as increase the accumulation of short-chain fatty acids (acetate, butyrate, and propionate). Additionally, it is important to highlight the ability of probiotics to produce antimicrobial compounds (hydrogen peroxide, lactic acid, bacteriocins, and short-chain fatty acids) able to inhibit the *Salmonella* proliferation or colonization in selected organs, such as ceca. [24,34,47,48,49,50,51,52,53]. Additionally, it has been reported that probiotics exert a reinforced effect of vaccines [44]. Table 2 summarizes some of the most representative effects observed through the probiotic supplementation in poultry.

### 4.3. Synbiotics

Synbiotics consist of a combination of prebiotics and probiotics. This strategy facilitates the implantation and survival of probiotics into the gastrointestinal tract due to their synergistic relationship [55]. Table 3 highlights some of the positive effects of the prebiotic–probiotic combination for poultry supplementation, focused on preventing and mitigating *Salmonella* infection.

According to the available reports, symbiotic-based strategies trigger some mechanisms involved in inhibiting *Salmonella* or lessen the clinical signs caused by *Salmonella* infection. These mechanisms may induce changes in the gastrointestinal microbiota composition, histopathological modifications in the intestine, binding to a variety of gram-negative organisms, or even through additive effects in the immune response mediated by antibodies. Effects observed using synbiotics are centered in the maintenance or improvement of the productive parameters of poultry due to the stimulation of productivity in broilers and laying hens, associated with the reduction of *Salmonella* sp. infections.

### 4.4. Postbiotics

In contrast to probiotics, postbiotics involve the use of non-viable bacteria or bacterial metabolic products such as inactivated cells, enzymes, exopolysaccharides, plasmalogens, organic acids, short-chain fatty acids, and peptides, mainly (but not exclusively) produced from lactic acid bacteria due to their multiple metabolic abilities [59]. Some of the benefits offered by postbiotics, when used in poultry, include the direct or indirect control of pathogens such as *Salmonella* and their negative effects (Table 4).

Experimental evidence confirms that the oral administration of postbiotics significantly reduced *Salmonella*-associated infections in poultry. The strategy can be based on the use of metabolic products from the controlled growth of lactic acid bacteria and yeast or their cell components to the use of bacterins produced by the target pathogen. Meanwhile, the main effects can be assessed by reducing *Salmonella* colonization in specific organs, the reinforcement of the gut microbiota, or stimulation of beneficial bacteria in the gut (such as *Lactobacillus*); some of the collateral effects include the improvement in nutrient absorption and growth performance. As reported by Wang et al. [47], the supplementation with albusin B increased the Preproendothelin-1 (PPET1) expression in the broiler jejunum, improving the amino acids and peptide uptake that increase the intestinal glucose and protein absorption (but not the mucosal development), inhibiting the adherence of pathogenic bacteria via lectin domain. Other strategies, such as supplementation with bacteriocins [61], can reduce *Salmonella* colonization by triggering the immune response in broilers. In this context, the bacteriocins most used as postbiotics exhibit cationic nature (at neutral pH), linked to the content of amino acids such as arginine, lysine, and histidine, which gives them the ability to bind to pathogens and compromise their cell integrity [62].

On the other hand, the use of bacterins also represents an alternative to prevent *Salmonella* infections in broiler chickens and significantly reduce infection signs, gross lesions, and mortality, besides enhancing the broilers’ performance [50]. Thus, the usage of postbiotics has provided benefits in poultry due to the stimulation of productivity in broilers and laying hens, associated with the reduction of *Salmonella* sp. infections [62].

### 4.5. Phytobiotics

Phytobiotics are plant-derived compounds or plant extracts that are used to improve the health status and productivity parameters of several animal species, including poultry. This involves the use of both herbs (non-woody and non-persistent plants) and spices (intensive smell and taste herbs) [63]. Most commonly used plants as phytobiotics include alfalfa [64], bergamot [65], peppermint [14], black cumin [66], chili [67], clove [68], oregano [69], cinnamon [70], and garlic [71], among others. It has been demonstrated that phytobiotics could enhance feed intake, stimulate the secretion of endogenous enzymes, reduce pathogens proliferation, improve the absorption of the nutrients, increase the carcass quality and muscle yield in broilers, and stimulate the immune system, among other effects [72]. Antimicrobial effects and microbiota modulation associated with phytobiotics also could involve cecal metabolic changes in poultry; nonetheless, the most relevant results observed related to *Salmonella* control are listed in Table 5.

## 5. Non-Feeding-Based Strategies

In addition to the feeding-based strategies, other alternatives have been extensively proved and implemented to reduce or control *Salmonella* infection in poultry. These strategies include the use of bacteriophages, in ovo applications, and vaccines.

### 5.1. Bacteriophages

After the discovery of bacteriophage viruses, independently by Frederik Twort in 1915 and Felix d’Herelle in 1917 [82], it was the same d’Herelle who, two years later, used bacteriophages for the treatment of children (3, 7, and 12 years old) with bacterial dysentery, observing recovery after 24 h of bacteriophages application. Later, several inward or in field bacteriophage trials were conducted until the discovery of penicillin in 1929 by Alexander Fleming, priming the onset of the antibiotic era. Although antibiotics displaced bacteriophages for the treatment of bacterial infections, research on bacteriophage therapy continued in the former Soviet Union, Poland, western Europe, and the United States [83]. Bacteriophage therapy is considered safe as bacteriophages are highly specific of a bacterial species or even of a particular strain, protecting the rest of the microbiota. Bacteriophages behave as “intelligent” or “active” drugs; they may be applied as a single dose, they replicate while there are still bacteria present, and decay in the same proportion as its target bacteria until both are cleared from the system. As bacteriophages are always present with their host bacteria, the immune system generally recognizes and tolerates them without being harmful to humans or animals, contrary to some antibiotics which may induce allergies. Replication of bacteriophages is easy and low-cost. Antibiotics may be co-administrated with bacteriophages, allowing synergic and most effective treatments. Bacteriophages are easy to manipulate genetically, so the improvement of the host range or changes in specificity may be generated. There are also some disadvantages of bacteriophage therapy, such as the need for specific phages for each strain or phage cocktails to avoid bacterial resistance, the possibility of neutralization by antibodies, inability to reach intracellular pathogens, and consumers acceptance, as it is a completely new approach for the control of bacterial infectious diseases [84]. Despite these disadvantages, bacteriophages are constantly coevolving with their hosts, and new bacteriophage isolates will be available in nature to overcome these problems.

Bacteriophages’ reproductive cycle has four modalities: lytic, lysogenic, pseudolysogenic cycles, and chronic infections [85]. In the lytic cycle, bacteriophages inject their nucleic acid into the bacterial cell, which biosynthetic machinery is sequestered by the virus to generate more viral particles, including the expression of cell wall lytic enzymes (endolysins) to free the particles to the environment. In the lysogenic cycle, bacteriophage (called temperate or lysogenic) nucleic acid is integrated into the DNA of the host bacteria and remains replicating with the bacterial genome as a prophage until the bacteriophage DNA is excised and the lytic cycle is induced. In the pseudolysogenic cycle, a part of the bacterial population enters the lytic cycle while another part remains lysogenic. Although pseudolysogenic or carrier-state bacteriophages are used as synonyms of pseudolysogeny, this latter state is commonly associated with the presence of plasmid-like prophages, reduced number of receptors in the bacterial host population, and mutations of superinfection immunity, thus allowing the presence of both bacteria and bacteriophage in the culture. A chronic infection cycle occurs when the bacteriophage is being reproductive inside the bacterial host without causing its lysis. Although lytic bacteriophages are the most useful tools for bacteriophage therapy, others showing the rest of reproductive cycles are also useful because of the presence of the cell wall lytic enzymes, the endolysin, responsible for cell wall lysis during bacteriophage release, and the viral associated peptidoglycan hydrolases (VAPGHs), which lyse the cell wall during nucleic acid injection into the bacterial cell [86].

#### 5.1.1. Bacteriophage Therapy

There is plenty of literature available on the search, identification, and characterization of lytic bacteriophages against *Salmonella* spp. Table 6 summarizes some of the reports related to bacteriophage application for the control of *Salmonella* infections in poultry.

All of these reports clearly support the great potential of bacteriophages against *Salmonella* in poultry and are the cornerstone to promote its production and commercial application in farms. Grant et al. in 2016 [15] and Wernicki et al. in 2017 [16], in comprehensive reviews of the use of bacteriophage therapy in poultry bacterial infectious diseases, also addressed the case of *Salmonella*. According to the reported literature, there are some highlights in the use of bacteriophages against *Salmonella*: (1) high titer of bacteriophages in single doses are better than repeated doses with low titer; (2) use of bacteriophages to prevent infections is poorly effective possibly due to the development of resistance; (3) efficiency of bacteriophage therapy depends on the adaptation of the bacteria to generate resistance; (4) bacteriophage cocktails are better than single bacteriophages; (5) synergy of bacteriophages with probiotics may enhance recovery by reducing mortality and spreading of bacteria; (6) although bacteriophages are considered as “generally regarded as safe” (GRAS) products to be used in food treatment, more studies on large production systems are needed to obtain FDA approval for its use in poultry farms.

#### 5.1.2. Phage Lytic Enzymes: Endolysins and Virion Associated Peptidoglycan Hydrolases (VAPGHs)

Another alternative derived from bacteriophages to treat or prevent *Salmonella* infections is the use of their peptidoglycan hydrolytic enzymes. There are two kinds of these enzymes (endolysins and Virion-associated peptidoglycan hydrolases) in bacteriophages [86]. Endolysins are the enzymes produced in the late stage of reproduction of bacteriophage and are responsible for the lysis of bacteria and Virion-associated peptidoglycan hydrolases (VAPGHs), which are responsible for degrading the bacterial cell wall to allow the injection of bacteriophage genetic material into the cell. Since both enzymes have peptidoglycan as a substrate, they behave as antibiotics because they can eliminate bacteria by lysis; therefore, they are considered enzybiotics, hydrolytic enzymes with antibiotic activity. Both endolysins and VAPGHs may be confirmed by one or more catalytic domains; endolysins also present a cell wall binding domain (CWBD) which is absent in VAPGHs. Endolysins of bacteriophage from Gram-negative bacteria usually contain a single catalytic domain and none, one or two CWBD, while endolysins related with Gram-positive bacteria may contain one or more catalytic domains or none, one or two CWBD. Endolysins are classified according to their enzymatic activity in (1) N-acetylmuramoyl-alanine amidases, which hydrolyze the amide bond between the N-acetyl-muramic in the glycan chain and the L-alanyl residues; (2) endo-β-N-acetylglucosaminidases, which hydrolyze the N-acetylglucosaminil-β-1,4-N-acetylmuramine acid linkage; (3) N-acetyl-β-muramidases, which catalyze the hydrolysis of N-acetylmuramoil-β-1,4-N-acetilglucosamine bond; (4) transglycosylases, which disrupt β-1-4 glycosidic bonds by forming a 1-6 anhydro ring in the N-acetylmuramic residue; (5) endopeptidases, which may hydrolyze both the tetrapeptide linked to the glycosil moieties or the pentapeptide intercrossing bridge [102,103]. The combination of some of these activities in endolysins and their CWBD give endolysins some specificity to the particular linkage they hydrolyze; however, as peptidoglycan has a generally conserved structure with few changes among bacterial taxons, endolysins may have a wider target range than bacteriophage host range, but not so wide to kill all the surrounding microbiota. Since endolysins are synthesized intracellularly previous to bacterial lysis, they require the presence of a holin, a pore-forming protein that allows the mobilization of the endolysin from the cytoplasm to periplasmic space, for it to reach the peptidoglycan.

To date, there are no commercially available products based on endolysin activity to the control or prevention of *Salmonella* infections in poultry, but there is a promising scenario on the utility of endolysins. Table 7 enlists some examples of endolysin applications in the poultry industry.

As shown in Table 7, in order for endolysins to show in vitro lysing activity against Gram-negative bacteria, they should be applied in combination with other proteins or compounds that allows the trespassing of the outer membrane, so the endolysin can reach the peptidoglycan in the periplasmic space. Friendly additives such as liposomes, which are already widely used in cosmetics and therapeutic applications, may help to avoid this problem [106].

To the best of our knowledge, to date, there are no reports on the use of VAPGHs to control any *Salmonella* spp. neither in vitro nor in animal experimental models, but they still have a potential for its use against *Salmonella* in poultry.

#### 5.1.3. What Is Still Needed to Consolidate Bacteriophage/Endolysin Therapy for *Salmonella* in Poultry?

There is a general acceptance in the industry of the safety of bacteriophage formulations for poultry by-products and other commercial feed susceptible to *Salmonella* contamination. However, there are still no regulations for the application of bacteriophages in animals or humans to treat infections. Bacteriophage/endolysin therapy differs from canonical pharmaceuticals in the personalized design; each pathogen isolate should be tested for the specific bacteriophages/endolysins–a tailor-made therapy. The European legislature coined the term Advanced Therapy Medicinal Products (ATMPs), which include personalized treatments as autologous somatic cell therapy and tissue engineering and may include bacteriophage/therapy [116]. The increasing number of clinical trials showing the efficacy of bacteriophages or their hydrolytic enzymes to combat multidrug-resistant *Salmonella* infections will certainly contribute to increasing its acceptance as pharmacological alternatives and will provide data to construct regulatory frames.

Bacteriophage, endolysins, or VAPGHs therapies are relatively young in the scenario of alternatives to control multidrug-resistant *Salmonella* infections, but multidisciplinary approaches to get better results with these therapies are emerging in the literature. The stability of each of them may be achieved by encapsulation into liposomes or nanoparticles that allow conserving their full activity in the body [106]. These approaches may also overcome the possibility that the immune system may promote the generation of specific antibodies, thus decreasing the effectiveness of bacteriophages or enzymes or generating immune reactions. Chemical modification such as PEGilation of bacteriophages or their lytic enzymes will also improve their in vitro stability and shelf lives.

Another interesting role for endolysins and VAPGHs is their use in the generation of bacterial ghosts. Bacterial ghosts are obtained from cells from the stationary phase of growth in which an inducible (for example, temperature-sensitive promoter) endolysin gene *E* from phage φX174 is expressed to lyse *Salmonella* cells. These cells are killed by lysis without disturbing the conformation of surface proteins, as it occurs in physical or inactivation methods to obtain vaccines [117]. This approach has been used to generate *Salmonella* Enteritidis ghosts with an overexpressed flagellin gene as a vaccine, which confers improved humoral and cell-mediated immune responses [118].

From the reports presented in this section, it is also evident that both bacteriophages and endolysins may present some specificity at serotype or genotype levels. Therefore, molecular genetic characterization of the *Salmonella* strains accompanying the analysis of efficiency of bacteriophage or endolysin therapies will also contribute to the better design of these tailor-made therapies.

### 5.2. Vaccines

The poultry industry usually implements strategies of surveillance and biosecurity at international, national, and farm levels to prevent *Salmonella* spread [119,120,121,122]. Among the health management protocols, vaccination represents the most efficient and cost-effective method to reduce the impact of clinical disease, maintain herd immunity, decrease the shedding and reduce both horizontal and vertical transmission of *Salmonella* in poultry flocks [19,123,124,125,126]. Additionally, poultry vaccination provides safer food products for consumers reducing the likelihood of food poisoning in humans [30,125,127,128]. The vaccination of layer and breeder flocks against *Salmonella* has a long history dating back to the second decade of the 20th century with the application of inactivated vaccines prepared from cultures of *Salmonella* Gallinarum [129]. Nowadays, the formulations of commercial *Salmonella* vaccines to the poultry industry are commonly based on strains of *S.* Enteritidis and *S.* Typhimurium [30,128,130,131,132]. *Salmonella* vaccines are divided primarily into three categories: live-attenuated, inactivated, and subunit vaccines [30,133]. An effective *Salmonella* vaccine should be safe, provide protection against different *Salmonella* serovars, and induce both humoral and cellular immunity to mediate long-term protection [134]. The type of vaccine to be used will depend on several local factors, including the type of production, level of biosecurity of the farm, local pattern of disease, status of maternal immunity, vaccines availability, method of administration, costs, and potential losses [135].

#### 5.2.1. Live-Attenuated Vaccines

Live *Salmonella* vaccines are given frequently to layer flocks and are based on a live attenuated variant of the pathogen, which presents an intrinsic balance between immunogenicity and reactogenicity [128,136,137]. Live-attenuated vaccines are administered parenterally or orally and have the ability to colonize the chicken’s gut, so they mimic the natural infection and stimulate cell-mediated, humoral, and mucosal immune responses [11,19,138,139]. The chicken intestinal innate immune system possesses several elements, including epithelial cells, monocytes, macrophages, dendritic cells, natural killer cells, neutrophils, cytokines, antimicrobial peptides, and nitric oxide, which limits the proliferation of pathogenic invading bacteria [140,141]. Through gut colonization capacity, *Salmonella* live vaccines have been used immediately after hatching when young poultry are immunologically immature. This promotes competitive exclusion; that is to say, some heterologous strains from *Salmonella* are no longer capable of colonizing the gastrointestinal tract, which results in an effective vaccination strategy [19,130,139,142].

#### 5.2.2. Killed or Inactivated Vaccine

Inactivated vaccines are based on killed/inactivated pathogens that cannot revert back to virulence [143]. *Salmonella*-killed vaccines are serovar-specific; that is, they are only effective only when the antigens between the vaccine strain and infecting pathogens are homologous [144]. Inactivated vaccines are administered by subcutaneous injection to breeders and layers flocks, increasing humoral immunity but not cell-mediated immune response [125,128]. Chickens immunized with inactivated *Salmonella* vaccines acquire a protective immunity to suppress *Salmonella* colonization in organs and reduce the shedding into feces [20,32]. However, lack of replication results in rapid elimination of the vaccine strain, which decreases the efficacy compared to live attenuated vaccines [142,145]. Frequently, the poultry industry prefers *Salmonella*-killed vaccines over the use of live vaccines for the level of biosecurity they offer [125]. However, it is necessary to consider that the intramuscular route of administration is time-consuming and is impractical when handling commercial poultry flocks [122]. For optimal protection, vaccination programs often include the sequential use of live attenuated vaccines followed by inactivated vaccines. This strategy induces high and uniform levels of protecting antibodies, which provide longer-lasting protection decreasing the chances of *Salmonella* outbreaks [122,146,147].

#### 5.2.3. Subunit Vaccines

The control of *Salmonella* in the poultry industry has relied heavily on live and inactivated vaccines [123,125,126,127]. However, over the last 30 years, advances in immunology, molecular biology, and recombinant DNA technology have allowed the identification and manipulation of the microbial components against which it is generated protective immunity, which has allowed to develop of vaccines that provide broader protection against multiple *Salmonella* serotypes [124,148]. Most subunit *Salmonella* vaccines are administered either intramuscularly or subcutaneously. Subunit vaccines contain one or more recombinant peptides/proteins or polysaccharides present in the structure of the target pathogen (rather than the complete pathogen) that, together with an appropriate adjuvant, elicit an appropriate humoral immune response [149]. Recent studies have shown that outer membrane proteins (OMPs), outer membrane vesicles (OMVs), and flagellin-proteins (FliC protein) of *Salmonella* are highly immunogenic in chickens [122,124,131,150]. Consequently, these molecules and other antigenic determinants have been used successfully for the expression and presentation of recombinant antigens [21]. Among the strategies developed in recent years for the delivery of recombinant antigens is biodegradable polymeric nanoparticles (NPs)-based vaccines [21,125,131]. This strategy has made it possible to develop subunit vaccines for oral administration, which allows directly delivering antigens to gut-associated lymphoid tissues (GALT), stimulating the proliferation of cell-mediated, humoral, and mucosal immune responses [21].

### 5.3. In Ovo Strategies

Chick embryo development has served as a model to understand the embryonic development in hens and other animals, and it has also been the basis to validate some in ovo approaches, useful to ensure the optimal development and productive behavior of hens and broilers. Embryo development takes 21 days on average and involves the process, in terms of formation and maturation of the gastrointestinal tract, from the formation of the alimentary tract stems (primitive streak) to the formation of villi and activation of some enzyme expression that prepare the young bird for the ingest of exogenous nutrients [151,152]. This process is essential to optimize the transitional period post-hatch that allows the enterocyte proliferation and the development of mucosal structures (including the mucosal layer), fundamental to protect the epithelial lining and the transportations of materials between the lumen and the brush-border membrane [151,153]. Gastrointestinal tract maturation is stimulated by feed intake, and it is crucial to the replacement of embryonic enterocytes by their matured counterpart. For this reason, early feed intake and gastrointestinal tract stimulation are crucial to avoid chicks to enter a starvation mode that limits the growth and development of the young bird, which could have repercussions through to market age [154,155,156,157], including a delay in reaching the market size, different gene expression patterns, and response to different stress conditions, among others [151,158,159,160]. Parallel to the maturation of the gastrointestinal tract, the process of gut microbiota occurs chiefly when exogenous nutrients are provided to the chickens. The gut microbiome is constituted by microorganisms that occur as “contaminants” of egg surfaces and their content, coming from the mother as well as the hatching environment; as a matter of fact, it has been reported that gut microbiota of chick embryos could be relatively rich, in terms of taxonomic diversity, since day 16 of incubation, with some species such as *Enterococcus*, *Micrococcus*, and *Bacillus* as predominant of that microbiota [161,162,163]. The composition and structure of embryos’ gut microbiota could be key in the early stimulation of the immune system of the bird, this being the principle of in ovo strategies. This technique was first used to improve the immune response against Marek’s disease [164,165] by the in ovo vaccination, reducing the lethality when birds were early exposed to the virus. From there, in ovo injection has been tested to dispense several types of biological compounds, including probiotics, nutrients, hormones, and immunostimulants, among others. Essentially, the principle of this technique was to provide nutrient solutions in the amniotic fluid of birds’ embryos (USA Patent #6,592,878 B2) [17,18], and it has been used to provide various types of nutrients, including carbohydrates (i.e., maltose, glucose), minerals (such as zinc), amino acids, prebiotics (mannanoligosaccharides, fructooligosaccharides), symbiotics, and vitamins (ascorbic acid), among others [166,167,168,169,170,171]. Main reported effects, obtained through in ovo administration of nutrients, include improvements in nutrient absorption, faster development of jejunum villus, immune system stimulation, increasing in enzymes and transporters expression, increased resistance against pathogens, and early development of digestive tract and muscle tissues [18,172], which, directly or indirectly, may contribute to control *Salmonella* infection or to mitigate its negative effects. Table 8 lists some of the reports related to *Salmonella* infection control based on the use of in ovo technique.

## 6. The –Omics as a Tool for *Salmonella* Strategies Development

In 1975, the concept of DNA sequencing was introduced; this technology was based on the incorporation of a deoxynucleoside triphosphate, fluorescently labeled and PCR primers, elements necessary for automated high-throughput DNA sequencing [189,190]. Later in the early 2000s, Life Science introduced its 454 Pyrosequencer; this technique was based on the preparation of a PCR emulsion, which allowed the detection of pyrophosphate released when a nucleotide was incorporated into the DNA chain resulting in light detectable in time real [191]. Subsequently, other technologies were developed, which gave rise to the platforms known as NGS (next-generation sequencing), which are based on which each DNA fragment is sequenced individually, and subsequently, the total sequences generated are analyzed [192]. Currently, there are other technologies for sequencing a single molecule in real time, carried out by Pacific Biosciences, which is based on the use of a nanostructured device, which allows sequencing in parallel, using a chip with thousands of nanoscale wells with an immobilized DNA polymerase linked to a primed DNA template for sequencing [193].

Thanks to the sequencing platforms, we can obtain information related to genomics, metabarcoding (16S and 18S amplicon sequencing), metagenomics (whole-genome sequencing), and transcriptomics. They are the main omics technologies that are currently used to investigate the genes contained in an organism, the microbiome present in different tissues, environments, the expression profile of genes in different conditions of an organism before a stimulus, and all this can be studied through these tools.

Within agricultural production, omics have been a very helpful tool; for example, the whole genome sequencing technology has allowed the identification of genes related to antimicrobial resistance, the study of the evolution of microbial strains, risk assessment, and epidemiology.

In 2004, the first draft of the genome of *Gallus gallus domesticus* was obtained; this provided information on its alleles and mutations related to its domestication and its subsequent specialization in meat-producing chickens and egg-producing chickens [194]. *Salmonella* in poultry has also been studied through NGS; for example, more than 30,000 *Salmonella* Enteritidis genomes from 98 countries have been studied for 71 years to try to predict by phylogenomics the spread of this pathogen through the world [195]. On the other hand, a comparative genomics analysis allowed evaluating the genotypic differences between *Salmonella enterica* serovar Gallinarum, revealing an open pangenome, where virulence factors, genomic islands, and antimicrobial resistance genes were identified. The information of this genome could help the identification of *Salmonella* strains and with this have fast and reliable diagnoses, in addition to the design of vaccines for the effective control against this pathogen [22].

The sequencing of amplicons and metagenomics has allowed us to evaluate the microorganisms present under certain conditions. An example of this is the study of the cecal luminal microbiota of laying poultry, which were supplemented with probiotics and challenged with *Salmonella* Typhimurium. The study revealed that the poultry with these supplements showed an abundance of *Ruminococcus, Trabulsiella, Bifidobacterium, Holdemania*, and *Oscillospira*, which indicates their role in maintaining intestinal health by reducing luminal pH and digestion of complex polysaccharides; however, this microbial diversity was not sufficient to reduce or eliminate the presence of *Salmonella* Typhimurium in the stool or invasion of other organs [196]. It has been proven that nutritious diets can help to gain body weight, promote the growth of villi in the intestine, as well as promote the increase of *Lactobacillus* in the ileum in broilers subjected to *Salmonella* Typhimurium [197]. In addition to nutritious diets, the effect of probiotics on the intestinal microbiota in egg-producing hens has been evaluated during *Salmonella* Typhimurium infection, finding that *Salmonella* negatively affects the diversity and abundance of many intestinal microbial genera such as *Blautia, Enorma, Faecalibacterium, Shuttleworthia, Sellimonas, Intestinimonas*, and *Subdoligranulum*, involved in important functions such as the production of organic acids and vitamins. Those treatments subjected to *Bacillus*-based probiotic supplementation improved their gut microbiota by balancing the abundance of genera displaced by *Salmonella* [196].

The transcriptomic study has allowed the identification of gene expression levels in response to different conditions or stimuli. An example of this is the work carried out by Wang and collaborators in 2019 [198], which compared the gene expression of the cecal tonsils of susceptible birds and resistance after *Salmonella* infection. Finding in resistant birds overexpressed genes related to the activation of the intestinal immune network for the production of IgA, which probably contributes to the protection and resistance of *Salmonella* infection [198]. Another report evaluates the differential expression of genes in birds infected with *Salmonella* Typhimurium, finding genes related to the immune response, IgA production, activation of the Toll-like signaling receptor pathway, and cytokine-cytokine interactions [11]. Cadena and collaborators identify genes over-expressed in *Salmonella* Heidelberg when it is subjected to different disinfectants, finding some related to virulence, pathogenicity, and resistance, allowing with this identification to make recommendations for the control of *Salmonella* [199].

Thanks to the NGS, the generation of data continues to increase, this information can provide the understanding and application of various strategies to reduce diseases caused by *Salmonella*, and this has a clear effect on the increase in commercial production. Thanks to NGS, we can evaluate some genes involved with antibiotic resistance in *Salmonella*, as well as track the spread of this disease throughout the world. We can also identify the microbiota present in the intestine, as well as evaluate the change in the proportion of this microbiota when it is attacked by an infectious agent; this change of proportions in the phyla has indicated a direct correlation with the health of the poultry. Thanks to the NGS, it has also been evaluated as a diet rich in nutrients and the use of probiotics and prebiotics favors the control of the growth of some pathogens and the reestablishment of microbiota of a healthy organism. The NGS have helped to identify some genes present in metabolic pathways responsible for the health of poultry; this can be used as a tool for detecting patterns of resistance to antibiotics, production of vitamins, and organic acids.

All these contributions have a direct impact on production with less cost for the implementation of strategies that reduce the presence of pathogens and are also useful in the generation and analysis of omics information that can result in the formulation of therapeutic strategies.

## 7. Conclusions

*Salmonella* infection is still one of the main challenges for the poultry industry, not only because of the disease and potential risk of mortality that it represents for the birds but also because of the losses and the reduction in efficiency caused by clinical or subclinical infection. Likewise, antibiotic resistance, associated with their uncontrolled use for both *Salmonella* control and as growth promoters, has led to the design and validation of accessible and profitable alternatives of natural origin to control *Salmonella* infection, prevent disease, and increase the productive performance of birds. Some emerging technologies to attend to these demands, supported in experimentation and scientific evidence, protect against *Salmonella* and other pathogens and improve the productive status of birds, either through individual use or by the synergy achieved by combining two or more of these antibiotic-free strategies. Meanwhile, advances in *omics* sciences have allowed a deeper understanding of the effects and mechanisms of using antibiotic-free approaches for *Salmonella* control in poultry. However, further knowledge is still needed to promote the use and commercialization of these valuable strategies to control *Salmonella* infections and better understand their functional potential.

## Figures and Tables

**Table 1:** Effects of dietary supplementation of prebiotics as a strategy to control *Salmonella* in poultry.

Target Species	Prebiotic	Experimental Procedure	Main Results	Ref.
60–65 w old White Leghorn hens	FOS	Birds housed at 27 ± 2 °C under a photoperiod of 16 h light: 8 h dark and supplemented with 0.1% of the prebiotic into a diet based on corn/soybean meal. Challenged against a nalidixic acid-resistant *S.* Enteritidis strain (2.4 × 10^8^ CFU)	FOS reduced fecal *S.* Enteritidis numbers and increased TLR-4, IFN γ, and IgA expression	[38]
Commercial meat-type broiler	Trehalose dihydrate	Broiler was supplemented with 5% *w/w* of the prebiotic and allocated at 22–30 °C (Humidity: 60 to 70%, dark-light 4/20 h photoperiod) and inoculated with *S.* Typhimurium (3.5 × 10^8^ CFU)	Trehalose increased the abundance of lactobacilli and suppressed the growth and inflammation caused by *S.* Typhimurium in the cecum	[39]
One-day-old Cobb broilers	2.6 Beta LevaFructan	Broilers were orally supplemented with the LevaFructan (100 gm on 1000 mL/0.5 mL per liter of drinking water) and maintained at 24 °C with a diet based on a balanced commercial ration. Challenge was performed by inoculation of *S.* Enteritidis (10^9^ CFU/mL) and a live lyophilized attenuated vaccine (*S.* Enteritidis Sm24/Rif 12/Ssq)	Prebiotics had a synergistic effect whit the vaccine on the decreasing of fecal isolation of *S.* Enteritidis	[40]
40 d old Cobb broilers	*Aspergillus* meal	Broilers were allocated on floor pens, fed with a commercial diet, and supplemented with 0.2% *w/w* of the *Aspergillus* meal. Challenge occurred by inoculation with *S.* Typhimurium (1.25 × 10^5^ CFU)	*Aspergillus* meal reduced *S.* Typhimurium horizontal transmission	[41]
Turkeys	*Aspergillus* meal	Turkeys were housed on floor pens, fed with a commercial diet supplemented with 0.2% *w/w* of *Aspergillus* meal, and challenged against *S.* Enteritidis (1.5 × 10^5^ CFU)	*Aspergillus* meal reduced *S.* Enteritidis colonization	[41]
Turkeys	Lactulose	Turkeys were fed with a corn and soybean meal diet, supplemented with lactulose (0.003 mL kg^−1^ body weight), and inoculated with *S.* Enteritidis (7.0 × 105 CFU).	*Salmonella* challenged Turkeys, but prebiotic supplemented increased weight gain	[42]

FOS—fructooligosaccharides; CFU—colonies forming units; TLR-4—toll-like receptor-4.

**Table 2:** Effects of dietary supplementation of probiotics as a strategy to control *Salmonella* in poultry.

Target Species	Probiotic	Experimental Procedure	Main Results	Ref.
One-day-old Cobb Broilers	*Lactobacillus acidophilus*, *Lactobacillus plantarum*, *Pediococcus pentosaceus*, *Saccharomyces cerevisiae*, *Bacillus subtilis*, and *Bacillus licheniformis*	Broilers were supplemented with a commercial probiotic-based preparation (1.0 × 10^9^ CFU/each strain), challenged against *S.* Enteritidis (0.5 mL, 109 CFU/mL), and inoculated with a live attenuated vaccine (*S.* enteritidis, strain Sm24/Rif 12/Ssq). Allocation was at 24 °C and feeding based on a commercial balanced ration	Probiotics exhibited a synergistic effect whit the vaccine and resulted in the decreasing of fecal isolation of *S.* Enteritidis	[44]
Broilers	*Lactobacillus acidophilus, Enterococcus faecium, Lactobacillus plantarum*, and *Lactobacillus casei*	Probiotic supplemented broilers (1 mg/4 L of drinking water of the commercial preparation) were challenged against *S.* Enteritidis (0.5 mL, 10^9^ CFU/mL). Diet consisted of a standard commercial starter concentrate	Probiotics prevented *Salmonella* infections in broilers	[50]
Hy-Line Brown layer hens	Poultry Star^®^ (*Enterococcus faecium, Pediococcus acidilactici, Bifidobacterium animalis*, and *Lactobacillus reuteri*)	Layers housed on floor pens and fed with stem-pelleted pullet starter and grower rations. Challenge consisted in the inoculation with *S.* Typhimurium PT 135 (10^6^ CFU per bird)	Probiotics enhanced the protection induced by vaccination with a live aro-A deletion mutant vaccine	[53]
Layer hens	*Bacillus subtilis* DSM 32324, *Bacillus subtilis* DSM 32325, and *Bacillus amyloliquefaciens*	Hens were allocated on floor pens and supplemented with the probiotic combination (1 g/kg of feed) to be challenged against *S.* Typhimurium (10^6^ CFU/mL)	Probiotic supplementation decreased *Salmonella* counts in feces	[46]
Layer hens (Hy-Line Brown)	*Bacillus amyloliquefaciens, B. licheniformis,* and *B. pumilus*	Hens were supplemented with the commercial preparation (454 g/ton of feed*)* and challenged against *S.* Enteritidis (3 × 10^7^ CFU/mL). Allocation consisted of floor pens and feed based on a basal diet, mash feed, and water offered ad libitum	Probiotics reduced the *Salmonella* recovery from layer ceca	[48]
Hy-Line Brown layer hens	*Bacillus subtilis* CSL2	Hens were housed on floor pens, fed with an antibiotic and additive-free basal diet, and inoculated with *S.* Gallinarum KVCC-BA0700722 (1 × 10^8^ CFU/mL)	Protective effects include improvement of bacterial diversity, enhanced metabolic activity and gut functionality, and reversal of the effects of *S.* Gallinarum infection	[54]
1 d-old Arbor Acres broilers	*Lactobacillus salivarius* Erya	Broilers were fed basal diet supplemented with *L. salivarius* Erya 10^7^, 10^8^ and 10^9^ CFU/kg of feed, vaccinated with attenuated infectious bursal disease virus vaccine and challenged against *Salmonella* Pullorum and exposed to aflatoxin B1 (AFB1).	L. salivarius degrade AFB1, enhanced antibody and IFN-γ production and lymphocite proliferaion, besides enhanced the resistance against *S.* Pullorum infection.	[51]
Broilers	*Bacillus subtilis*	Broilers were supplemented with the commercial probiotic preparation (0.2% *w/w* of feed) and challenged against *S*. Enteritidis (0.2 mL of 1.0 × 10^5^ CFU/)mL. Housing consisted of floor pens and fed in a basal diet	Probiotic improved the immune response of *Salmonella*-infected broilers	[49]

CFU—colonies forming units.

**Table 3:** Effects of dietary supplementation of synbiotics as a strategy to control *Salmonella* in poultry.

Target Species	Synbiotic	Experimental Procedure	Main Results	Ref.
Hy-Line Brown laying hens	Commercial probiotic mix (*Enterococcus faecium, Pediococcus acidilactici, Bifidobacterium animalis, Lactobacilus reuteri*) + fructooligosaccharides	Symbiotic supplementation (20 g/1000 birds/day) of hens allocated on floor pens, orally-infected with *S.* Typhimurium PT 135 (10^6^ CFU per bird), and vaccinated against *Salmonella*	The synbiotic enhanced the immune response in vaccinated hens, inhibiting *Salmonella* shedding pattern	[53]
Hy-Line pullets	Commercial symbiotic: *Bacillus* + mannooligosaccharide	Pullets were supplemented with the symbiotic (0.075% *w/w* of diet formulated with 113 g/ton of amprolium) and challenged against a Nalidixic acid-resistant *S.* Enteritidis strain (3 × 10^6^ CFU)	The symbiotic-supplemented birds exhibited reduced colonization of ceca and ovary with *S.* Enteritidis	[56]
Hy-Line W-36 laying hens	*Bacillus amyloliquefaciens, Bacillus licheniformis,* and *Bacillus pumilus* (250 ppm) and yeast cell wall (mannan and β-glucan, 250 ppm)	Synbiotic were mixed with the commercial feed and supplemented to hens challenged against a nalidixic acid-resistant *S.* Enteritidis strain (7 × 10^7^ CFU)	The synbiotic reduced the counts of *S.* Enteritidis from ceca	[48]
Dekalb White female chicks	*Bacillus subtilis* C-3102 (250,000 CFU/g) and 0.05% of yeast cell walls	Birds were allocated into floor cages and fed with a nonmedicated ration based on corn and soybean. A nalidixic acid-resistant strain of *S.* Enteritidis was inoculated to chicks (2.1 × 10^9^ CFU)	A significantly lower abundance of *Salmonella* was found in the cecal microbiota of supplemented birds	[57]
Cobb broilers	Commercial synbiotic (*Saccharomyces* sp. and *Lactobacillus* sp.)	Birds feeding includes a commercial broiler feed supplemented with the commercial synbiotic. Broilers were allocated into battery cages and inoculated with *S.* Enteritidis (1 × 10^9^ CFU)	*Salmonella*-challenged Broilers challenged, but synbiotic-supplemented increased the weight gain and maintained immunity response compared to its unsupplemented counterpart	[58]
COBB Avian48 broilers	*Lactobacillus rhamnosus* HN001 and *Pediococcus acidilactici* MA18/5M (7 log CFU, and fructans from Agave tequilana (4.5%)	Broilers were allocated into floor pens and fed with an antibiotic-free diet supplemented with the synbiotic. The challenge consisted of inoculation with *S.* Typhimurium PT 135 (10^5^ log CFU per bird)	*S.* Typhimurium was inhibited in synbiotic-supplemented broilers and resulted in a decrease in the intensity and frequency of histopathological injuries	[55]

CFU—colonies forming units.

**Table 4:** Effects of dietary supplementation of postbiotics as a strategy to control *Salmonella* in poultry.

Target Species	Postbiotic Strategy	Experimental Procedure	Main Results	Ref.
Hy-Line W-36 laying pullets	Fermentation-based postbiotic from *Saccharomyces cerevisiae*	Birds were housed in rooms, fed with a standard commercial starter and grower, postibiotic-supplemented (1.5 kg/MT in the starter diet, 1.0 kg/MT in the grower diet), and challenged against *S.* Enteritidis (1.0 × 10^6^ CFU/mL)	Postbiotics reduced *S.* Enteritidis concentrations in the ceca	[59]
Broilers	Semi-purified Albusin B from *Ruminococcus albus* 7 (2.5 g/kg)	Broilers were housed into pens under controlled conditions and fed with a basal diet based on corn/soybean meal. Challenge was performed by *Salmonella* spp. Inoculation (6.15 log CFU/g)	*Salmonella* colonization was reduced in postbiotic-supplemented broilers as well as nutrient absorption was improved	[60]
43-day-old broilers	Bacteriocin L-1077 from *Lactobacillus salivarius* 1077 (12.5 mg/L of drinking water)	Birds were inoculated with an *S. enterica* serovar Enteritidis 0.2 mL suspension of 10^11^ CFU/mL for 3 days while feed and water were offered ad libitum	Bacteriocin supplementation reduced *Salmonella* counts	[61]
Broilers	*Salmonella* Enteritidis bacterin from an autogenous *Salmonella* Enteritidis (0.2 mL of/bird)	Broilers were fed with a standard commercial ration and challenged against *S.* Enteritidis (0.5 mL containing 10^9^ CFU/mL)	Bacterin inactivated *S.* Enteritidis and avoided pathogenic infection in broilers	[50]

MT – Metric Ton; CFU - colonies forming units.

**Table 5:** Effects of dietary supplementation of phytobiotics as a strategy to control *Salmonella* in poultry.

Target Species	Phytobiotic	Procedure	Main Results	Ref.
Cobb broiler chicks	Garlic extract	Five consecutive days of garlic extract orally administered (200 mm/)mL 24 h later of *Salmonella* infection	In vitro inhibition of *S.* Typhimurium*. S.* Papuana*, S.* Inganda*, S.* Kentucky*, S.* Enteritidis*, S.* Heidelberg*, S.* Molade*, S.* Tamale*, S.* Labadi (Minimum inhibitory concentration of 40–100 mg/)mL. Decrease in mortality and increase in body weight in supplemented chickens and challenged against *S.* Typhimurium	[73]
Cobb X Cobb broilers	Capsaicin	Inclusion of purified capsaicin (10 ppm), capsaicin oleoresin in finisher diet of *S.* Enteritidis challenged birds (5 or 20 ppm), or prophylactic use for prevention of *S.* Typhimurium infection (5 or 20 ppm)	Reduction in *S.* Enteritidis colonization in liver/spleen and ceca when used purified capsaicin. Inclusion of 5 ppm reduced *S.* Enteritidis colonization in ceca and decreased cecal lamina propia thickness. Prophylactic use of capsaicin induced resistance to *S.* Typhimurium	[74]
20 d old Ross X Ross broilers	Plant-derived trans-Cinnamaldehyde (TC) and Eugenol (EG)	Birds were supplemented with TC (0.5 or 0.75%) or EG (0.75 or 1%) and inoculated with *S.* Enteritidis on day 8	Both TC and EG reduced *S.* Enteritidis colonization of the cecum after 10 d of infection. TC did not affect feed intake and body weight; meanwhile, body weight was lower in EG supplemented birds	[75]
One-day-old male Cobb × Cobb broilers	Essential oil blend (carvacrol, thymol, eucalyptol, lemon)	Essential oil blend was administrated in drinking water to chicks (0–7 and 35–42 day), and a half of birds were challenged against *S.* Heidelberg	An inclusion of 0.05% of the essential oil blend reduced *S.* Heidelberg colonization in crops of challenged birds, but no effect was observed when 0.025 or 0.015% concentrations were used. The essential oil also lowered feed conversion ratio and improved weight gain	[76]
1 d old male broiler Cobb 500 chicks	Phytogenic feed additive based in essential oils (Carvacrol, thymol, and cinnamic aldehyde)	Chickens were supplemented with 0.5 or 1% of the additive and monitored for the total bacterial count in bed samples on day 42	Total bacterial count in bed samples was reduced by 1% of inclusion of the feed additive, and total erythrocyte counts and hemoglobin content increased, while lymphocyte counts decreased	[77]
Ross 308 chickens	Commercial phytobiotic based on a mix of essential oils Intebio (garlic, lemon, thyme, and eucalyptus)	Administration of the phytobiotic mixture since 1 d old and challenge against *S.* Enteritidis at day 19	One day post infection, genes AvBD10, IL6, IL8L2, CASP6, and IRF7 were upregulated, and their expression was lower at day 23 in the infected birds. Intebio did not involve a pronounced change in microbiota but an earlier suppression of inflammatory reaction	[78]
Cobb broiler chickens	Propyl propane Thiosulfonate derived from garlic (PTS-O)	Feed inclusion of PTS-O (45 or 90 mg/kg of diet)	Both concentrations of the compound resulted in lower number of copies (log10) of ileal *Salmonella* sp., crop Enterobacteria, and *Escherichia* coli. Feed–gain ratios were improved as well as ileal villus height, width and surface area, mucosal thickness, and muscular layer thickness	[79]
Dekalb hens	Capsaicin	Two levels supplementation of the capsaicin (18 and 36 ppm). Hens were challenged against *S.* Enteritidis on day 25	*Salmonella* liver and spleen invasion was reduced when hens were supplemented with 36 ppm of capsaicin. Capsaicin also increased the deposition of red pigment in egg yolk	[67]
Ross 308 broiler chicks	Sanguinarine, oregano.	Birds were supplemented with the phytobiotics and their combination con probiotic strains and challenged on day 2 against *S.* Typhimorium	Phytobiotics improved growth performance and gut health through the mitigation of the negative effect of the disease	[80]
Ross 308 broiler chicks	Commercial mixture of 7 plant extracts (oregano, eucalyptus, thyme, garlic, lemon, rosemary, and sweet orange)	Three presentations of phytobiotic mixture (Mix-Oil Mint, Mix-Oil Liquid, Sangrovit Extra) were administrated to birds infected with *S.* Typhimorium	Supplemented and *Salmonella* challenged birds exhibited growth performance and improvements in meat characteristics comparable with their counterpart treated with the antibiotic avilamycin	[81]

**Table 6:** Studies on bacteriophage therapy for *Salmonella* infections in poultry.

Target Species	Description ^b^	Phage Application ^c^	Results	Ref.
One-day-old chicken	Oral challenge *S.* Enteritidis PT4 10^8^ CFU/bird	Single oral application of phage cocktail (CNPSA1, CNPSA3, and CNPSA4) 10^11^ PFU	Reduction in 3.5 orders of magnitude of CFU of *S*. Enteritidis PT4 per gram of cecal content	[87]
6-week-old chickens	Oral challenge *S*. Gallinarum 5 × 10^8^ CFU/mL	Bacteriophage CJø01 as food additive at 10^6^ PFU/kg	Reduction from 30% to 5% of mortality	[88]
One-day-old chickens	Challenge with *S*. Enteritidis by oral gavage (0.25 mL) 9 × 10^3^ CFU/chick	Cocktail of 4 bacteriophages (CB4φ) from commercial broiler houses; cocktail of 45 bacteriophages (WT45φ) from wastewater treatment plants; 10^8^ PFU/chick	Short time (24–48 h) prevention of colonization. No long-term effect	[89]
36-day-old chickens	Challenge with *S*. Enteritidis, 1 mL of 10^8^ CFU/mL	Cocktail of three bacteriophages (151, 25, and 10) against *S*. Enteritidis, *S*. Hadar, *S*. Typhimurium, by oral gavage, 10^9^ and 10^11^ PFU/ml	Reduction of 2–4 log units of *S*. Enteritidis and *S*. Typhimurium after 10^11^ PFU/ml	[90]
33-day-old quails	Oral challenge, 100 mL *S.* Enteritidis, 1.2 × 10^9^ CFU/ml	Single *Salmonella* lysing phage (PSE), 10^9^ PFU/mL 100 µL by oral beverage for 2 days	100% clearance of *S*. Enteritidis from tonsils after 6 h of treatment	[91]
One-day-old chickens	Oral challenge, 0.5 mL of *S*. Typhimurium, 2.4 × 10^5^ or 7.9 × 10^5^ CFU/mL	Bacteriophage cocktail (S2a, S9, S11), 10^6^ PFU/bird at days 4–6 and 8–10 of age. Supplementation with commercial probiotic	10-fold reduction of *S*. Typhimurium in ileum, ceca, liver, and spleen. Synergism with the probiotic	[92]
Ten-day-old chickens	Oral challenge, *S*. Enteritidis 9.6 × 10^5^ CFU/mL	Cocktail of three bacteriophages from sewage system, 10^3^ PFU by coarse spray or drinking water, 24 h prior to bacterial challenge	Reduction from 5.67 (control) to 4.04 (aerosol) and 4.25 (drinking water) log_10_ CFU/mLof *S*. Enteritidis	[93]
One-day-old chickens	Oral challenge, 2.5 × 10^5^ CFU/mLof *S*. Enteritidis	Cocktail of three phages () 10^8^ PFU//mLdose by aerosol at 6 days of age. Probiotic supplementation	Reduction of 100% of mortality	[94]
One-day-old chickens	Oral challenge, *S*. Enteritidis, 5 × 10^8^ CFU/mL	Bacteriophage CJ07, 10^5^, 10^7^, 10^9^ PFU/g, 21 days after challenge	Higher titers reduced replication of the pathogen in the digestive tract	[95]
Three-day-old, specific pathogen-free chickens	Oral challenge, *S*. Typhimurium 10^5^ CFU/animal (10 times the lethal dose)	Cocktail of three bacteriophages (UAB_Phi20, UAB_Phi78, UAB_Phi87) lytic against *S*. Enteritidis and *S.* Typhimurium. 10^10^ PFU/animal. Treatment from day-1 to 15 post infection	Reduction in 2–4.4 log_10_ of *S.* Typhimurium. Repeated administration of the cocktail maintained bacteriophage titers by 10^4^–10^5^ PFU/g cecal content	[96]
14-day-old broiler chickens	No challenge, prevalence evaluation in a large-scale study (more than 69,000 chicks)	SalmoFREE^®^, commercial cocktail of six bacteriophages in drinking water, 1 × 10^8^ PFU/mL	Reduction to 0% of prevalence in cloacal swabs, PCR detection	[97]
70-day-old broiler chickens	Feed challenge, *S*. Enteritidis 10^7^ CFU/g food	Bacteriophage KCTC 12012BP, 10^8^ PFU/g food	Reduction of the prevalence of *S.* Enteritidis in cloacal swabs, liver/spleen samples, and ceca	[98]
Two-week-old chickens	Feed challenge, *S*. Typhimurium, 1 × 10^8^ CFU/g food	Bacteriophages STP4-a, 10^9^ PFU/g food; pre-treated 7 days before bacterial challenge, treated 14 days after bacterial challenge	Pretreatment eliminated *S*. Typhimurium; treatment reduced bacterial counts in 2 log_10_ units	[99]
One-day-old chickens	Oral gavage challenge, *S*. Enteritidis, 6 × 10^6^ CFU	Bacteriophage cocktail (BRM 13312, BRM 13313, BRM 13314) 6.8 × 10^10^ PFU/broiler. Early treatment (days 6–10 post-infection), late treatment (days 31–35 post-infection)	Later treatment was more effective, reducing by 1.08 log_10_ CFU/g cecal content.	[100]
One-day-old broiler chickens	Oral challenge, 0.1 mLat 10^8^ CFU/,mL *S*. Kentucky	Oral administration, 0.1 mL at 10^8^ PFU/mL	Reduction in 1.76 to 2.6 log_10_ units. No significant difference if bacteriophages were administrated before or after challenge	[101]

Modified from Wernicki et al., 2017; CFU—colony-forming units; PFU—plaque-forming units.

**Table 7:** Endolysin applications against *Salmonella*.

Target Pathogens	Endolysin Application	Main Results	Ref.
*S*. Typhimurium LT2, *A. baumannii* 2, *P. aeruginosa* PAO1, *P. fluorescens* 7A, *Shigella sonnei* ATCC 25931, *E. coli* O157:H7 CECT 4782, *Cronobacter sakazakii* CECT 858, *Pantoea agglomerans* SA5634, *Enterobacter amnigenus* CECT 4878, *Proteus mirabilis* SA5445, *Salmonella bongori* SGSC 3100, *Klebsiella oxytoca* ATCC 13182, *Yersinia enterocolitica* SA5429	Recombinant endolysin Lys68 (phage from *S.* Enteritidis), 2 µM with EDTA and organic acids as permeabilizers	Broad spectrum of activity in the presence of malic acid as permeabilizer	[104]
Clinical or reference strains of *Pseudomonas aeruginosa*, *Acinetobacter baumannii*. *Klebsiella pneumoniae*, *Escherichia coli*, *Salmonella typhi*, *Staphylococcus aureus*, *Staphylococcus haemolyticus*	Recombinant endolysins LysAm24 (phage from *A. baumannii*), LysECD7 (phage from *E. coli*), LysSi3 (phage from *S.* Typhi), 5–15 µg/mL	Broad spectrum of specificity against all strains except those from the genus *Staphylococcus*, which showed total or partial resistance	[105]
*Salmonella* Typhimurium LT2, *Escherichia coli* DH5α	Recombinant endolysin BSP16Lys (phage from *S*. Typhiumurium) loaded into liposomes to trespass outer membrane barrier	BSP16Lys loaded into liposomes were active against *S*. Typhimurium and *E. coli*	[106]
*Streptococcus pyogenes* ATCC19615, *Bacillus subtilis* ATCC 12711, *Bacillus* sp., *P. aeruginosa* ATCC 27853, *E. coli* ATCC 25922, *S*. Typhimurium DDBCC1001, *Proteus* sp., *K. pneumoniae* ATCC 13883	Purified endolysin Lys46_30_, from a lysogenic bacteriophage SPP1 against *Bacillus subtilis*	Lytic activity against Gram-negative pathogens tested	[107]
*S. enterica* ATCC13076, *E. coli* ATCC35150, *Shigella flexneri* CMCC51572	Recombinant Endolysin LyS15S6 (phage *Salmonella-virus-FelixO1*) administrated with ε-poly-L-lysine as outer membrane permeabilizer	Reduction in 4.19, 3.18, and 3.00 log_10_ units, respectively	[108]
Multidrug-resistant strains of *S*. Enteritidis, *S*. Typhimurium, *S*. Agora, *S.* Indiana, *S.* Anatum, *S.* Dublin	Recombinant endolysin LysSE24 administrated with outer membrane permeabilizers	Broad spectrum of activity mainly against multidrug resistance *Salmonella*	[109]
*A. baumanii*, *Enterococcus faecalis*, *Escherichia coli*, *Klebsiella pneumoniae*, *Pseudomonas aeruginosa*, *Salmonella* Enteritidis, *S*. Typhimurium, *Staphylococcus aureus*	Recombinant endolysis LysSS (phage from *S*. Enteritidis)	Broad spectrum of antibacterial activity. Active without a permeabilizer additive. Different minimal inhibitory concentrations for *A. baumannii* genotypes	[110]
Several strains of *A. baumannii*, *Salmonella* spp., *Pseudomonas aeruginosa*, *Enterococcus faecium*, *Enterococcus faecalis*, *Staphylococcus aureus*.	Recombinant endolysin Abtn-4 (phage from *Acinetobacter baumannii*), 5 µM. No permeabilizer added	Reduction of Gram-negatives in more than 3 log_10_ units, active against Gram-positives. Reduction of biofilm formation for both Gram-positives and Gram-negatives	[111]
Clinical isolates from *S.* Enteritidis, *S.* Infantis, S. Typhimurium, S. Dublin, S London, *A. baumanii*, *Enterobacter* spp., *P. aeruginosa*, *K. pneumoniae*, *Campylobacter jejuni*	Individually tested recombinant endolysins (100 µg/)mL LysAm24 (phage from *A. baumannii*), LysAp22 (phage from *A. baumannii*), LysSi3 (phage from *S*. Infantis), LysSt11 (phage from *S*. Typhimurium), LysECD7 (phage from *E. coli*)	All tested isolates showed broad spectrum of activity	[112]
*Pseudomonas aeruginosa*, *S*. Typhimurium, S. Enteritidis, *S*. Gallinarum *Klebsiella pneumoniae*, methicillin-resistant *Staphylococcus aureus* (MRSA), methicillin-sensitive *S. aureus* (MSSA)	Combined use of recombinant endolysin RL-Lys and holin RLH-Lys (phage from *P. aeruginosa*)	The holin allows the entrance of the endolysin into the periplasmic space showing a broad-spectrum activity	[113]
*S*. Typhimurium, *S*. Enteritidis, S. Paratyphi A, *S*. Paratyphi B, *Shigella dysenteriae*, *S. boydii*, *E. coli*, *Lysteria monocytogenes*, *S. aureus*	Recombinant LysSp1 (phage from *S*. Typhimurium) 1–10 µg/mLin the presence of EDTA	Reduction in 1–6 log_10_ units, broad spectrum of specificity	[114]
*S*. Typhimurium NBRC 12529, *S*. Typhimurium FHC, *S.* Anatum, *S.* Braenderup, *S*. Derby, *S*. Enteritidis FHC, *S*. Hadar, *S*. Litchfield, *S*. Stanley, *Escherichia coli* NBRC 3301 (K-12), *E. coli* BW25113, Enterohemorrhagic *E. coli* O91:H-*1, Enterohemorrhagic *E. coli* O157: H7, *Pseudomonas aeruginosa* NBRC 13275, *Staphylococcus aureus* NCTC8325, *Listeria monocytogenes* No.180	Recombinant endolysin LysSTG2 (phage from *S*. Typhimurium), 2–800 µg/mLin the presence of chloroform/Tris-HCl (spectrum activity assay) or slightly acidic hypochlorous water (SAHW; 40 mg/L chlorine, pH5.5) for biofilm assay in *S*. Typhimurium	Broad spectrum of activity on Gram-negative bacteria. Synergy with SAHW in biofilm assays	[115]

**Table 8:** In ovo alternatives to control *Salmonella* infection in poultry.

Target Species	Delivered Compound	Experimental Procedure	Main Results	Ref.
SPF Ross 308 broilers	Vaccine	Vaccination *Salmonella* flagellin to 18 day old embryonated eggs	Elevated pro-inflammatory chIL-6 and chIL-8 cytokine transcript levels 24 h post-vaccination. High titers of FliC-specific antibodies 21 day post-hatch	[173]
Cobb 500 embryonated eggs	Probiotics	Inoculation with a 3 × 10^11^ CFU/mL suspension of *Lactobacillus acidophilus, L. fermentum*, and *L. salivarius* in the air cell of 18 d embryonated eggs. *S.* Enteritidis inoculation 2 day after hatching	No decrease (*p* > 0.05) in *S.* Enteritidis colonization of chick ceca.	[174]
Coob 500 broiler fertil eggs	Prebiotics	In ovo injection of Raffinose (1.5, 3.0, and 4.5 mg in 0.2 mL of aqueous diluents) into the air sac of 12 day embryonated eggs	Increase of the villus height, the villus height–crypt depth ratio (*p* < 0.05), and the expression levels of CD3 and chB6	[175]
Ross 308 hatching eggs	Prebiotics and synbiotics	Administration, in the air chamber at 12 day of incubation, of inulin, Bi^2^tos, inulin, and *Lactococcus lactis* subs. *lactis* or Bi^2^tos and *Lactococcus lactis* subs. *lactis*	Modulation of central and peripheral lymphatic organ development (cortex/medulla ratio in the thymus, development of cortex in bursal follicles, and germinal center’s formation in the spleens), especially through the use of symbiotics	[176]
Broilers’ embryonated eggs	Prebiotic	Commercial egg injector system (Inovoject^TM^) to apply a dextrin solution (18% maltodextrin, 10% potato extract dextrin) containing iodinated casein (80, 240, 720, or 2160 µg/)mL	Improvement in hatchability and early growth attributable to iodinated casein in combination with Dextrin. No differences in *Salmonella* colonization after chicks were challenged	[177]
Ross 308 *Salmonella* free hatching eggs	Probiotic	In ovo injection, into the air cell, at 18 day incubation with 0.1 mL of a commercial probiotic suspension (7 × 107 CFU/mL in PBS). After hatching, chicks were challenged against *S.* Enteritidis (Se) (8 log CFU)	Reduction in the number of Se colonized chicks since day 1 post hatching. Reduction of Se colonization in the alimentary tract of chicks	[178]
Fertile broiler eggs	Probiotic	Probiotic administration, at 18 day of incubation, of Marek’s vaccine + one of three *Bacillus subtilis* strains (ATCC 6051, ATCC 8473, ATCC 9466)	Results regarding hatching were strain-dependent; however, probiotic strains reduced the bacterial counts (total aerobes and coliforms) in the ileum and ceca	[172]
White Leghorn hens	Immune lymphokines	On day 18 of embryogenesis, eggs were injected into the amnion with Immune (ILK) and nonimmune (NILK) Lymphokines. Post-hatch, chicks were orally challenged against *S.* Enteritidis (5 × 10^4^ CFU)	In vitro bactericidal activity was higher, and organ invasion with *S.* Enteritidis decreased in ILK-treated chicks. Hatchability was not affected, although ILK-treated chicks were 1 g lighter than NILK-treated ones (*p* < 0.05)	[179]
Broiler embryonated eggs	Probiotic	Injection, at 18 day of incubation, of an undefined and anaerobically grown competitive exclusion culture into the air cells or beneath the inner air cell membrane	Evident resistance to *S.* Typhimorium of chicks challenged at day 7 post hatch	[180]
Broiler embryonated eggs	Probiotic	A competitive exclusion culture consisting of several species of unrevealed bacteria injected either the air cell or body of the 18 day of incubation embryos	Injection in the body proper resulted in losing almost all the hatchability. Hatchability was reduced and mortality during the first week increased in air cell injected embryos. No effects on *Salmonella* infection were observed when chicks were challenged 1 day after hatching	[181]
Ross embryonated eggs	Probiotic	18 d incubating eggs were inoculated using cecal microbiota (total or diluted) and *Lactobacillus salivarius* into the inner air sac	Maximum hatchability observed was 65%. 2-d chicks were challenged against *S.* Enteritidis. Liver and cecum colonization was not reduced in the in ovo inoculated chicks	[182]
18-d White Leghorn 15I_5_ × 7_1_ embryonated eggs	Probiotic	Eggs were inoculated after 18 d of incubation with a commercial probiotic (FloraMax^®^-B11) through injection into the amnion. After hatching, chickens were orally infected with *S.* Enteritidis	Probiotic administration did not affect hatchability but increased body weight during first 7 d, increased the villi surface area in the ileum and reduced the presence of lactose-positive Gram-negative bacteria, as well as reduced the incidence of *S.* Enteritidis	[183]
Broiler embryonated eggs	Antibiotic	Gentamicin was administered at 18 d of incubation into the amnion. At hatching, chicks were gavaged with a commercial Competitive Exclusion Culture (MSC^®^. 0.2ml, 1 × 10^8^ UFC/)mL and challenged against *S.* Typhimurium	A cumulative effect was observed by the in ovo application of Gentamicin and the supplementation with the Competitive Exclusion Culture at hatching in ceca colonization with *S.* Typhimurium	[184]
Ross × Ross 708 fertile eggs	Probiotic	18 d of incubation eggs were inoculated with an *Enterococcus faecium*-based commercial probiotic (Galli-Pro Hatch) at three concentrations	Hatchability was not affected, and live performance in the first 21 days were improved as well as yolk absorption and intestinal and spleen morphology	[185]
Ross 308 broiler embryonated eggs	Probiotic	Injection was performed at 17.5 d of incubation for the inoculation of two probiotic strains (*Enterococcus faecium* and *Bacillus subtilis*). Chicks were orally challenged against *S.* Enteritidis 4 days post hatching	Probiotic administration, at a dose up to 10^9^ CFU/egg) not only reduces but also eliminates the presence of *Salmonella* in broilers	[186]
Cobb 500 fertile eggs	Probiotic	On day 18 embryonic day, eggs were injected into the air cell with a commercial probiotic (Primalac W/S. *Lactobacillus acidophilus, Lactobacillus casei, Enterococcus faecium*, and *Bifidobacterium bifidum*) using three different concentrations	Hatchability, feed intake, and feed conversion ratio were not affected by the probiotic administration. The expression of immune-related genes in the ileum and cecal tonsils were modulated	[187]
Broiler fertilized eggs from a commercial breeder	Immune response stimulation	18 d old embryos were injected in the amnion with CpG oliodeoxynucleotides (CpG-ODN) and orally infected with *S.* Enteritidis at day 10 post-hatch	Colonization of *S.* Enteritidis in ceca was reduced greater than 10-fold in comparison to placebo birds. CpG-ODN stimulates innate immune responsiveness of birds heterophils	[188]

## Data Availability

Not applicable.
